# Three-dimensional digital imaging analysis of the palatal bone thickness for orthodontic mini-implant insertion – determination of the safe zone and angulation

**DOI:** 10.1186/s12903-024-05229-y

**Published:** 2024-11-28

**Authors:** Hanna Schubert, Ragai Matta, Anna Seidel, Werner Adler, Manfred Wichmann, Marco Kesting, Rainer Lutz

**Affiliations:** 1 Department of Oral and Cranio-Maxillofacial Surgery, University Hospital Erlangen of Friedrich-Alexander University Erlangen-Nürnberg (FAU), Glückstraße 11, 91054 Erlangen, Germany; 2 Department of Prosthodontics, University Hospital Erlangen of Friedrich-Alexander University Erlangen-Nürnberg (FAU), Erlangen, Germany; 3 Department of Medical Informatics, Biometry and Epidemiology, Friedrich-Alexander University Erlangen-Nürnberg (FAU), Erlangen, Germany

**Keywords:** Three-dimensional, Palate, Cone-beam computed tomography, Temporary anchorage devices, Orthodontic anchorage procedures

## Abstract

**Background:**

In order to successfully perform orthodontic mini-implant procedures successfully for the subsequent anchoring of orthodontic appliances, it is crucial to know the palatal bone thickness. This is usually assessed using two-dimensional radiographs. The purpose of this study was to use a three-dimensional digital imaging measurement method to provide information on palatal bone volume and bone thickness and to make recommendations on the optimal and safe insertion site and angle for palatal mini-implants.

**Methods:**

For this observational, cross-section study, pre-existing cone beam computed tomography scans of 184 patients were converted into 3D Standard Tessellation Language (STL) models of the maxilla. The area between the canine and the first molar was divided into 6 regions of interest (ROIs), three on the left side of the palate and three on the right side. The bone thickness of the palate was analyzed volumetrically and linearly while simulating different mini-implant insertion angles of 0°, 10°, 20° and 30° degrees relative to the palatal surface.

**Results:**

Among the ROIs, the greatest mean bone thickness was observed in the region of the first premolars with a mean distance (MD) of 10.44 ± 2.53 mm and decreased from anterior to posterior (MD: 3.44 ± 1.16 mm). The highest volume (Vol) values were also measured in the anterior palatal region (Vol: 1127.26 ± 483.91 mm^3^), while there was also a decrease in the posterior region (Vol: 394.36 ± 180.22 mm^3^). Regarding the simulated palatal mini-implant insertion sites, the greatest bone thickness was found in the anterior region, at the level of the canines with an angle of 0° (MD: 12.25 ± 3.75 mm). In the more posterior region, at the level between the first and second premolars, the greatest bone thickness was observed at an angle of 30° (MD: 7.93 ± 3.81 mm). Gender differences were found.

**Conclusion:**

This clinical study showed that the paramedian region at the level of the first premolar is the safest site for orthodontic mini-implant placement as evaluated by three-dimensional measurements. The results showed that implant insertion angle, gender and age are important aspects to consider when planning and inserting orthodontic palatal mini-implants.

**Trial registration:**

N.a.

**Supplementary Information:**

The online version contains supplementary material available at 10.1186/s12903-024-05229-y.

## Background

Orthodontic palatal mini-implants have proven to be an effective method of bony anchorage for orthodontic appliances to correct malocclusions in adolescent and adult patients. The reason why anchorage is so important in orthodontic treatment is to prevent unwanted tooth movement, whereas elsewhere teeth can be repositioned by the appliance [1]. There are several ways to achieve sufficient anchorage for intended tooth movement in orthodontic treatment. Traditional anchorage options include extraoral headgear, intraoral transpalatal arches, brackets and fixed retainers cemented to the teeth (Nance buttons) [2–4]. However, the disadvantage of these “old school” devices is that the results achieved are highly variable and depend on the patient’s cooperation and the specific and often complex design to be adapted to the patient [3, 5]. Recently introduced and more modern alternatives are temporary anchorage devices (TADs), such as orthodontic mini-implants. They are implanted directly into the bone and can therefore be used for a variety of treatment options when high forces are required, such as distalisation and mesialisation of molars, midline correction, extrusion of impacted teeth and even transversal expansion of the maxilla in the case of a transversal deficit [6–9]. The use of orthodontic mini-implants has become increasingly popular because they have been shown to have success rates as high as 80–100%, are easy to place, and are independent of patient compliance [1, 10, 11]. The most common reason for treatment failure with orthodontic mini-implants is loosening or loss of the implant during therapy due to poor oral hygiene [1, 12, 13]. The failure rate (loss, fracture, mobility, etc.) of TADs during orthodontic treatment ranges from 0.00 to 40.8%, with an overall mean failure rate of 13.5% [14]. If orthodontic mini-implants fail, a second implant can be placed in the same site after 3 months [1]. An implant diameter of 2–2.3 mm and a length of 7–11 mm is required to avoid the risk of loosening, and attention should be paid to rotational forces during implant loading [15–17]. Other important factors for a TAD implantation are primary stability, patient age and bone density [18, 19]. In addition, bone supply and mucosal condition are discussed as having a direct effect on mini-implant anchorage [12].

However, despite the many benefits, implantation requires a surgical procedure that can lead to intraoperative accidents [20]. Risks associated with implantation include damage to anatomical structures such as nerves, and the traumatization of the adjacent tooth roots [2, 18, 21].

The position of the orthodontic mini-implants should be carefully planned, as it will influence the outcome of the treatment and should be chosen according to the treatment objective. Excessive angulation of the implant or inadequate planning may result in tooth injury [16].

The “T-zone” or “safe zone” described by Becker et al. takes into account bone thickness and quality through linear, two-dimensional measurements at 25 points on the palate [18]. In the “T-zone”, the bone thickness was greater than 6.5 mm, allowing safe placement of mini-implants. It extended from the first premolars to the contact point between the first and second premolars. The paramedian zone of the palate at the contact point between the canines and the first premolars was considered unsuitable because of the large interpatient variation [18].

### Objectives

The aim was to determine the mean anatomical characteristics of the “T-zone” and to enable the safest placement of orthodontic mini-implants. This study aimed to evaluate the volume and linear thickness of the local palatal bone for mini-implant placement by analyzing CBCT scans of a large cohort of patients in order to obtain a detailed and precise three-dimensional analysis. The objective of the present study is to provide recommendations regarding bone thickness for clinicians, in both linear and angulated forms within the typical insertion area for mini implants. Through this anatomical analysis, the potential risks related to the placement of mini implants can be minimised, ultimately optimising the treatment with mini implants. This study aimed to determine whether there is an ideal insertion angle relative to the surface of the palatal bone and optimal positions for orthodontic mini-implants in terms of bone height. Furthermore, it also aimed to determine whether the safe “T-zone” changes with age or gender.

## Materials and methods

### Study design and study cohort

This retrospective clinical observational, cross-section study was conducted in accordance with the Declaration of Helsinki on medical protocol and ethics and was carried out after approval by the local ethics committee (IRB number 350_18B). Recommendations for reporting were followed using the STROBE guideline [22].

**Fig. 1:**
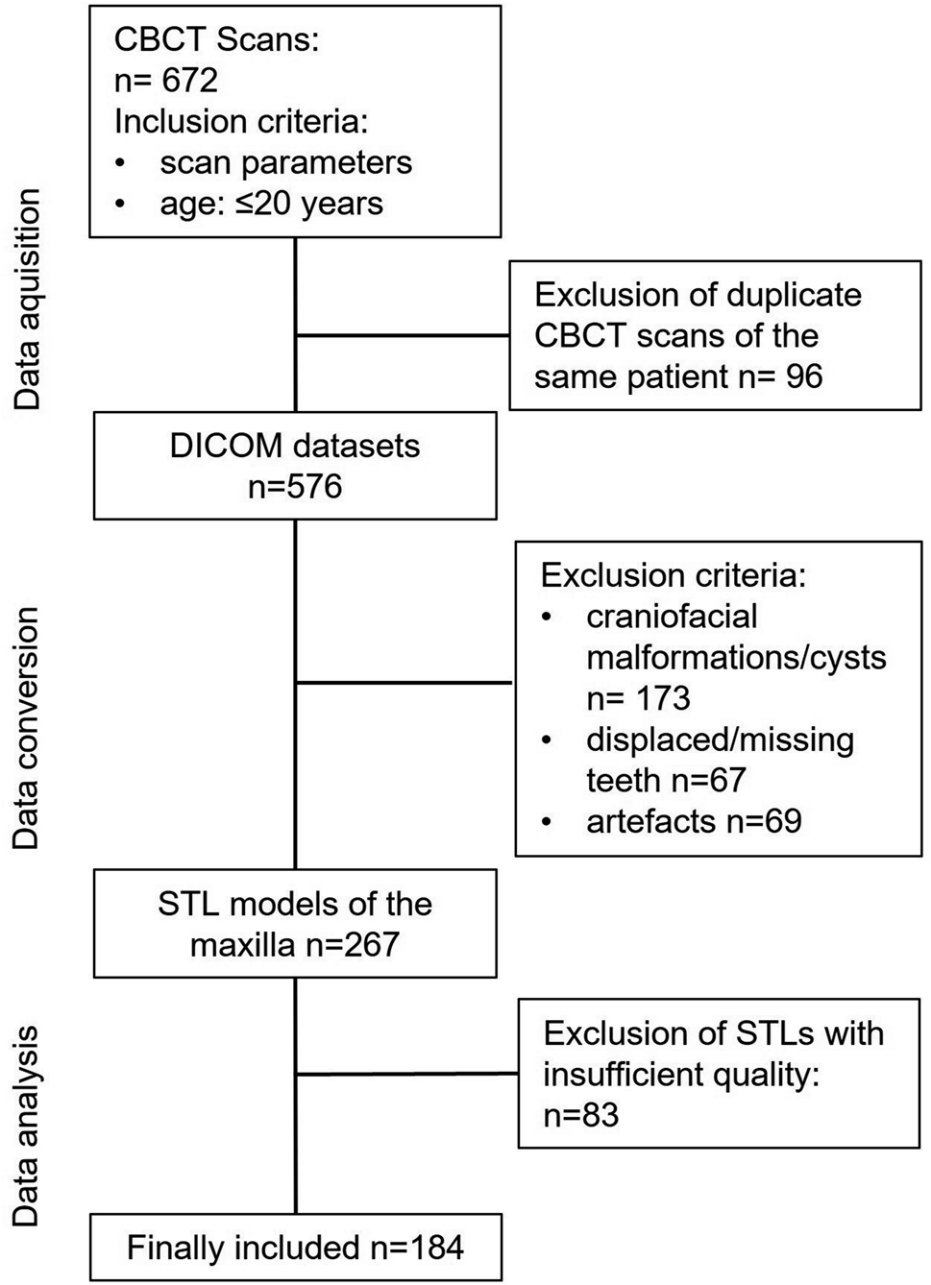
Overview of the exclusion procedure during the evaluation process

Cone beam computed tomography (CBCT) scans of patients acquired between March 2010 and August 2018 at the Department of Prosthodontics and the Department of Maxillofacial Surgery, University Hospital Erlangen, Friedrich-Alexander University, Erlangen-Nürnberg, Germany, were reviewed for eligibility. All CBCT scans were acquired with a justified medical indication and not for the purpose of this study. A total of 672 CBCT scans were available and viewed (Fig. 1). Patients with complete dentition and no history of orthodontic treatment were included. Furthermore, only CBCT scans with sufficient acquisition quality and without artefacts were included. Patients with craniofacial malformations such as unilateral or bilateral cleft lip and palate or asymmetries were excluded. Patients with missing teeth, unduplicated or duplicated teeth, displaced teeth, fractures, orthognathic surgery and/or metal restorations in the maxilla were excluded. Patients with pathological processes in the maxilla or nasal cavity, such as cysts, were also not considered. CBCT scans were acquired with the following parameters 0.3 × 0.3 × 0.3 mm voxel, 120 kV, 5 mA and 8.9 s acquisition time. Finally, data from 184 CBCT scans of 184 patients (62 female and 122 male patients) were included in the study.

### DICOM data conversion

**Fig. 2:**
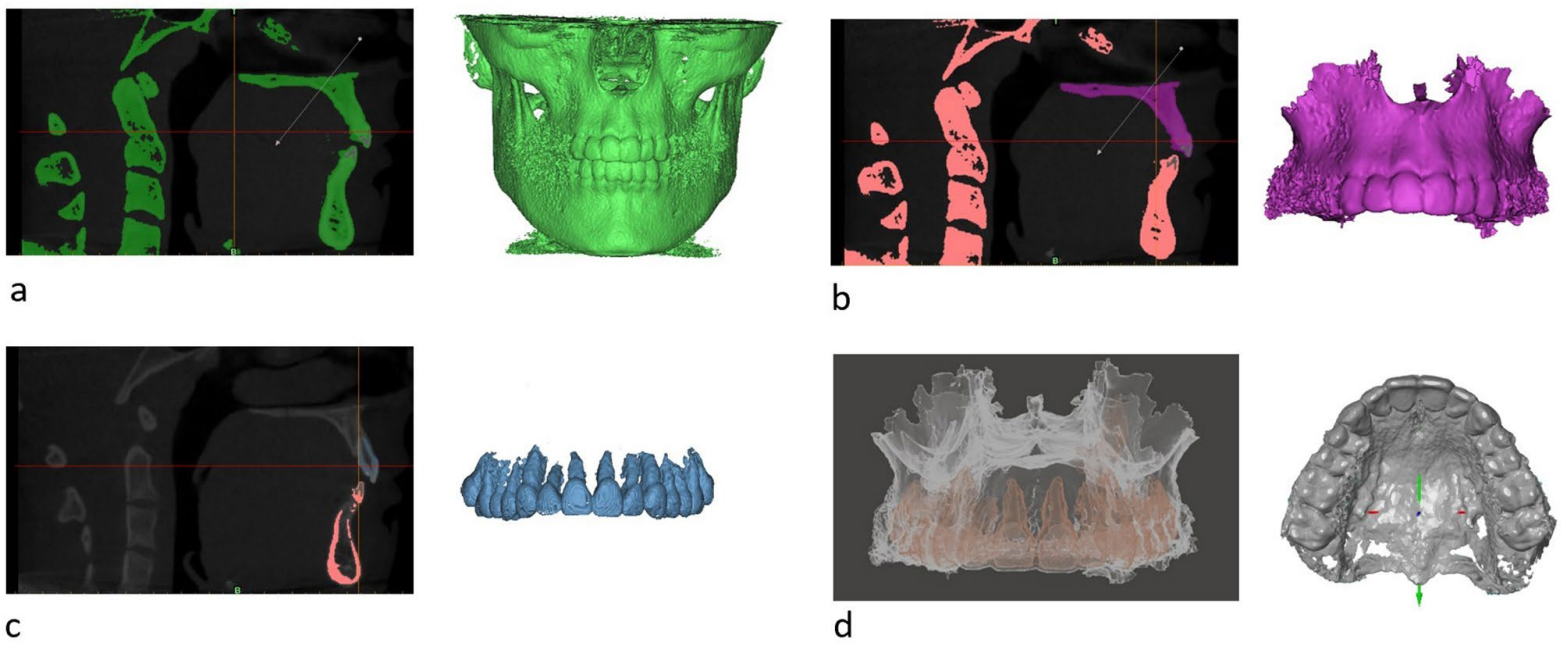
Illustration of Data conversion. (**a**) complete CBCT scan in DICOM format (green); (**b**) segmentation of the maxilla (magenta); (**c**) separate selection and thresholding of the teeth (blue); (**d**) matching process: STL model of the maxilla with teeth and nerve canal (**a-c** software Mimics Innovation Suite; **d**: software Meshmixer)

**Fig. 3:**
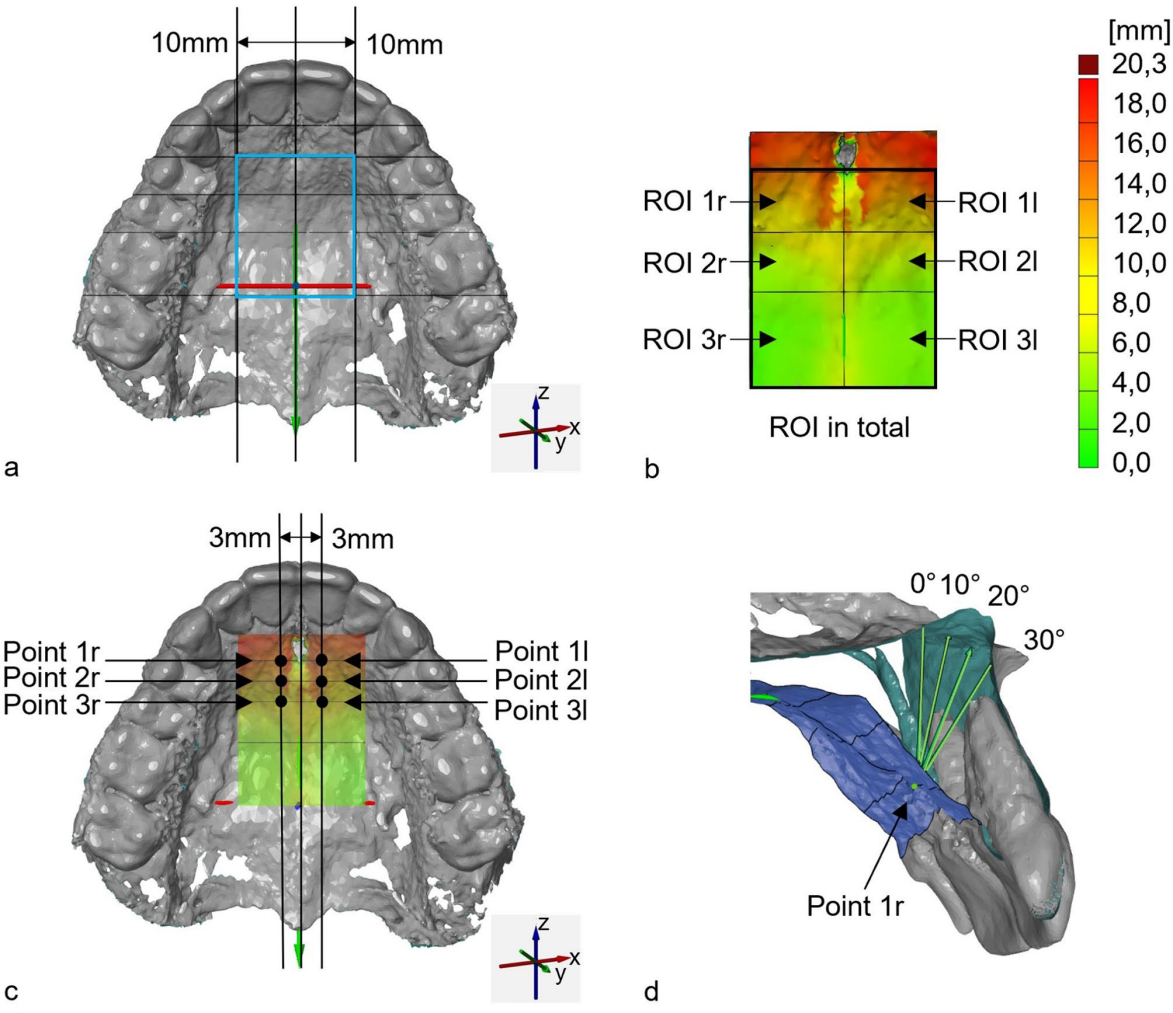
Illustration of the measurement procedure. (**a**) Illustration of the region of interest (ROI, blue) with its 10 mm paramedian boundary and subdivision of individual regions 1–3 along the teeth as a reference; (**b**) ROIs 1–3 of the right (r) and left (l) side with color-coded distance map representing the bone thickness in mm; (c) Illustration of the set points 3 mm paramedian, simulating possible implant insertion sites; (**d**) visualization of the bone thickness measurement (green arrows) in point 1r with angulations of 0°, 10°, 20° and 30° (blue = palatal bone to oral cavity; turquoise = bony floor of nasal cavity; gray = tooth roots of the incisors)

To generate digital 3D models of the maxilla for further analysis, the CBCT scans were converted from Digital Imaging and Communications in Medicine (DICOM) datasets into the desired Standard Tesselation Language (STL) files using medical DICOM data analysis software (Mimics Innovation Suite 19.0, Materialise, Leuven, Belgium). For this purpose, an individual grey level measurement was performed for each DICOM file, which served as an orientation to determine the individual threshold for the segmentation of the maxillary hard tissues. As CBCT grey values do not correlate exactly with Hounsfield units (HU), manual thresholding was performed to obtain optimal results for the STL models [23, 24]. In order to standardize the conversion, all thresholds were chosen in the range of 50-2000 HU and all HU measurements were taken through the same anatomical structures (tongue, palate and air) (Fig. 2a). All voxels with a gray value above the specified threshold then formed the 3D model of the maxilla, which was exported as a STL file (Fig. 2b). In order to minimize inaccuracies, the segmentation protocol recommended by Forst et al. was followed during the conversion [25]. This included both automated segmentation and human refinement to achieve the most accurate result. As the STL format only provides information on surfaces (including the nerve canal), this file does not contain information on the tooth roots within the maxillary bone. Therefore, in a second step, another threshold was set to perform an individual segmentation of the teeth (including their roots) and convert them into an additional STL file (Fig. 2c). To preserve the orientation of both models, the maxillary and dental STL models were merged in a 3D processing tool (Meshmixer, Co. Autodesk, San Francisco, USA) (Fig. 2d) [26]. As the exact same orientation was imported, there was no inaccuracy in the matching process and the two meshes were automatically merged. The result was a 3D STL model of the maxilla with complete anatomical structures such as tooth roots and the nerve canal (Fig. 3d).

### Digital 3D evaluation

The evaluation of the generated 3D models was carried out using industrial computer-aided design (CAD) evaluation software (GOM Inspect Suite 2022, Carl Zeiss GOM Metrology GmbH, Braunschweig, Germany). First, the STL models were imported and a coordinate system was created with orientation according to anatomical structures: the Y-axis through the anterior and posterior nasal spine, the X-axis according to the distal fissure of the first maxillary molars and the Z-axis was set perpendicular to the occlusal plane (Fig. 3a). The thickness of the maxillary palatal process was measured in a defined palatal region that forms the anterior two-thirds of the hard palate and the floor of the nasal cavity [27]. The region of interest (ROI) was defined along the medial and paramedian sutura palatina of the maxilla from the first premolar to the first molar. The ROI was divided into 6 sections (Fig. 3b) representing the “T-Zone” described by Becker et al., of the left and right palate with a section of the first premolar, second premolar and first molar [18].

#### Linear and volumetric calculation of bone thickness in the ROI

Each ROI section was selected and the analysis area (AA) [mm^2^] and the mean distance (MD) of all measuring points of the bone height in [mm] were measured. A segmentation method was used to calculate the volume of the bone thickness, and a 3D comparison was made between the palate bony surface and the bony floor of the nasal cavity in each segment. The volume (Vol) [mm^3^] was calculated as the integrated distance, by multiplying the area (AA) by the mean distance (MD) in each segment (Table 1). The results were recorded descriptively (Table 2) and the MD values were displayed in a color-coded distance map (Fig. 3b).

#### Linear bone thickness measurement with angulations

For linear bone thickness assessment, the area at the level of the first premolars was examined in more detail, as this is generally the area selected for mini-implant placement. Six points were defined as shown in Fig. 3c: The lateral orientation was 3 mm paramedian of the median palatal suture, and the anterior-posterior orientation was defined along the teeth of the maxilla: between the canine and the first premolar (point 1r/1l), in the middle of the first premolar (point 2r/2l), and between the first and second premolars (point 3r/3l) (Table 1). Wilmes et al. recommend an angle of approximately 10°-30° perpendicular to the occlusal plane during insertion, so the bone thickness of the palate was measured for each point at an angle of 0°, 10°, 20° and 30° relative to the palatal surface [16]. The software assessed the thickness of the bony palate in millimeters [mm], calculated as the distance between the palatal surface oriented towards the oral cavity and the coronal surface, which is the bony floor of the nasal cavity (Fig. 3d).

**Table 1:** Description of the area to be analysed and the measured variables

Variables	Description
Vol /Volume	Total bone volume (integrated distance) for each ROI in [mm^3^]
MD / Mean distance	Mean linear bone thickness measured at all points within the ROI in [mm]
Mean	Mean value of bone thickness measured at each defined point in [mm]
AA / Analysis area	Surface area of analysis in [mm^2^]
Areas of investigation	Description
ROI 1r/1l	Area of analysis at the level the premolar; r = right, l = left
ROI 2r/2l	Area of analysis at the level the second premolar; r = right, l = left
ROI 3r/3l	Area of analysis at the level the first molar; r = right, l = left
Point measurement with an angle of 0°, 10°, 20° and 30°	Description
Point 1r/1l	Measurement point at the level between the canine and the first premolar and 3 mm paramedian of the sutura palatina; r = right, l = left
Point 2r/2l	Measurement point at the level of the first premolar and 3 mm paramedian of the sutura palatina; r = right, l = left
Point 3r/3l	Measurement point at the level between the first and second premolars and 3 mm paramedian of the sutura palatina; r = right, l = left

### Statistical analysis

Statistical analysis was performed using the statistical software R V4.3.1 (R Core Team 2023, R: A language and Environment for Statistical Computing, Vienna, Austria). We visualized age dependency of bone thickness at different angles using spline smoothing. The evaluation involved the development of a several of mixed linear regression models with volume and MD as dependent variable, respectively. Either the angle or the ROI were the independent variables (evaluation of “difference between angles” or “difference volume”). When angles were examined, we added the point type (point 1r/1l, point 2r/2l or point 3r/3l) as independent variable when the measurements between points differed systematically. To account for the fact that the bony palate is our statistical unit and measurements of different angles or ROIs of the same palate are not really independent of each other, mixed models with a random intercept were used. This allowed for the use of all measurements (different ROIs, angles, and points) in the model without the need to calculate average values for each observation. Normal distribution of the residuals, an assumption for the validity of the model, was visually inspected and non-normal distribution could not be excluded. However, Schielzeth et al. showed that linear mixed-effects models are robust against violations of assumptions [28]. Additionally, we validated significant results of the mixed model by averaging statistically dependent measurement values and performing nonparametric tests (Mann-Whitney U test and Wilcoxon signed-rank test, respectively, where appropriate). These results are provided in the supplementary file.

In additional models age group, sex, teeth, and side were added to the list of independent variables, where “teeth” was used to indicate whether the upper incisors extend into the ROI. Because the influence of teeth and angle was very different when examining point 1r/1l in comparison to points 2r/2l and 3r/3l, a model for point 1 (model point 1r/1l) was calculated and a model for points 2 and 3 (model point 2r/2l/point 3r/3l). The regression coefficient, the corresponding 95% confidence interval (95% CI) and the p-value were calculated. The significance level was set at *p* < 0.05.

## Results

### Patient demographics

A total of 184 CBCTs (62 female, 122 male patients; age 16.03 ± 3.78 years) were examined. The patient cohort was divided into three age groups as performed in a previous study by Chhatwani et al. [29]. The patients were divided into three age groups: 4–12 years old (*n* = 33; male *n* = 20; female *n* = 13), 13–16 years old (*n* = 42; male *n* = 24; female *n* = 18) and the last age group, 17–20 years old (*n* = 109 patients; male *n* = 78; female *n* = 31).

### Volumetric and linear evaluation at ROI

#### Volumetric evaluation at ROI

Bone volume was found to be highest in the anterior region ROI 1r /ROI 1l (1116.31 ± 458.42 mm^3^ and 1127.26 ± 483.91 mm^3^) and decreased significantly (*p* < 0.001) towards the posterior region 403.80 ± 158.54 mm^3^ for ROI 3r and 394.36 ± 180.22 mm^3^ for 3l respectively (Table 2).

Vol measurements were compared between the subdivided regions (ROI 1r-3l). The Vol in the ROIs decreased significantly from anterior to posterior (*p* < 0.001). No significant difference was found between the sides (*p* > 0.05). There was also a gender difference in bone volume (*p* < 0.001). Female patients had lower values than male patients of the same age (regression coefficient = -144.93). No significant difference was found in the relation to age (*p* = 0.90; *p* = 0.92) (Table 3).

#### Linear evaluation at ROI

Regarding the mean linear measurements, the highest bone thickness was found in the region of the first maxillary premolar in ROI 1r/ROI 1l (10.44 ± 2.53 mm and 10.40 ± 2.52 mm, respectively). Bone thickness decreased significantly from anterior, ROI 1r/ROI 1l, to posterior, ROI 3r/ROI 3l (*p* < 0.001). In the region of the second premolar at ROI 2r/ROI 2l, the mean bone thickness was approximately half that of the anterior region (5.67 ± 2.05 mm and 5.68 ± 2.09 mm, respectively). The smallest mean distances were measured in ROI 3r/ROI 3l (3.52 ± 1.13 and 3.44 ± 1.16 mm, respectively) (Table 2).

**Table 2:** Descriptive results of vol and MD: the mean values (Mean), standard deviation (SD), minimal (Min) and maximum (Max) values, skewness and kurtosis of measured volume in mm^*3*^ and of the mean distance in Mm

		Mean [mm^3^] (95%CI)	SD [mm^3^]	Min [mm^3^]	Max [mm^3^]	Skewness	Excess Kurtosis
VOL	ROI in total	3973.07 (3763; 4182.2)	1447.37	1303.15	10049.87	0.991	1.683
	ROI 1r	1116.31 (1050.07; 1182.54)	458.42	311.15	3662.80	1.791	5.871
	ROI 1l	1127.26 (1057.34; 1197.18)	483.91	192.91	3661.15	1.894	6.588
	ROI 2r	468.82 (431.48; 506.16)	258.40	96.06	1394.96	1.409	2.190
	ROI 2l	465.59 (427.98; 503.2)	260.29	82.31	1908.39	1.710	5.018
	ROI 3r	403.80 (380.89; 426.71)	158.54	136.94	894.09	0.740	0.078
	ROI 3l	394.36 (368.32; 420.4)	180.22	134.23	1563.64	2.117	9.237
		Mean [mm] (95%CI)	SD [mm]	Min [mm]	Max [mm]	Skewness	Excess Kurtosis
MD	ROI in total	6.80 (6.54; 7.07)	1.84	3.05	12.21	0.399	-0.169
	ROI 1r	10.44 (10.07; 10.81)	2.53	4.69	17.61	0.139	-0.130
	ROI 1l	10.40 (10.04; 10.77)	2.52	3.29	16.89	-0.093	-0.021
	ROI 2r	5.67 (5.38: 5.97)	2.05	1.53	11.85	0.550	-0.074
	ROI 2l	5.68 (5.37; 5.98)	2.09	1.23	11.37	0.501	-0.128
	ROI 3r	3.52 (3.35; 3.68)	1.13	1.48	6.89	0.567	-0.165
	ROI 3l	3.44 (3.28; 3.61)	1.16	1.26	7.18	0.627	-0.167

MD measurements were compared between the subdivided regions (ROI 1r-3l). The ROI decreased significantly from anterior to posterior (*p* < 0.001). No significant difference was found between the sides (*p* > 0.05). There was also a gender difference in bone thickness (*p* < 0.001). Female patients had lower values than male patients of the same age (regression coefficient = -1.12) (Table 3). There was no significant difference in age (*p* = 0.14, *p* = 0.11) (Table 3).

**Table 3:** Mixed regression models: variation in volume and MD depending on various influencing factors (age group, sex, teeth, side, ROI) as further independent variables with regression coefficient, 95% confidence interval and p-value

VOL [mm^3^]					Coefficient	95% CI					*p*-value
Parameter		Groups		Number [n]		Lower Limit		Upper Limit			
intercept					1095.60	993.88		1197.32			< 0.001
age group [years]		Reference: 4–12		33							
		13–16		42	6.64	-94.36		107.64			0.90
		17–20		109	4.61	-83.95		93.18			0.92
sex		Reference: male		122							
		female		62	-144.93	-213.89		-75.98			< 0.001
teeth		Reference: no teeth		79							
		teeth		105	122.12	56.58		187.68			< 0.001
side		Reference: left		184							
		right		184	0.57	-28.04		29.18			0.97
ROI		Reference:1r/1l		184							
		2r/2l		184	-654.58	-689.62		-619.54			< 0.001
		3r/3l		184	-722.70	-757.74		-687.66			< 0.001
MD [mm]					Coefficient	95% CI					p-value
Parameter						Lower Limit		Upper Limit			
intercept					9.94	9.24		10.64			< 0.001
age group [years]		Reference: 4–12		33							
		13–16		42	0.52	-0.18		1.22			0.14
		17–20		109	0.51	-0.13		1.14			0.11
sex		Reference: male		122							
		female		62	-1.12	-1.61		-0.63			< 0.001
teeth		Reference: no teeth		79							
		teeth		105	0.73	0.28		1.18			0.002
side		Reference: left		184							
		right		184	0.04	-0.10		0.17			0.61
ROI		Reference: 1r/1l		184							
		2r/2l		184	-4.75	-4.92		-4.58			< 0.001
		3r/3l		184	-6.94	-7.11		-6.77			< 0.001

### Linear punctual measurements

The variability in bone quantity was shown for different points and angles. The results of the bone thickness measurements at one insertion angle showed high individual variation at anterior points 1r and 1l, especially at angles of 20° and 30°. As seen in the ROI area analysis, bone thickness decreased from anterior to posterior. The highest bone thickness was found at point 1r with 12.25 ± 3.75 mm at 0° and 11.81 ± 4.27 mm at 10°. Points 2r/2l and 3r/3l had the highest bone thickness at an angle of 30°. Values of 10.79 ± 3.84 mm and 11.0 ± 3.78 mm were measured at point 2r/2l, while lower values of 7.93 ± 3.81 mm and 7.88 ± 3.65 mm were measured at point 3r/3l.

**Fig. 4:**
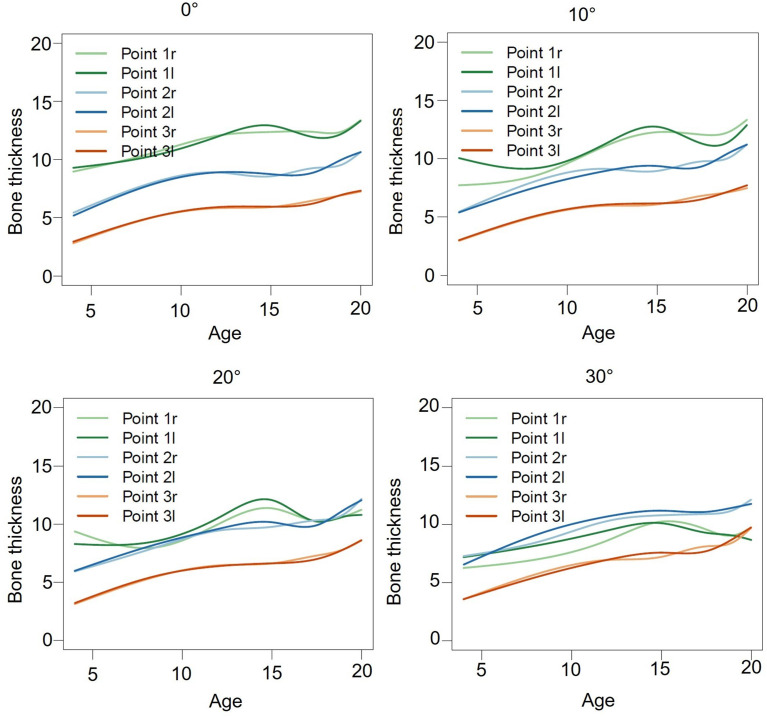
Graphical visualization of the age dependency of bone thickness at different angles of 0°, 10°, 20° and 30° by using smoothing splines. x-axis = age; y-axis = bone thickness in mm; r = right; l = left

The lowest value was measured at point 3l at an angle of 0° (6.34 ± 2.59 mm) (Fig. 4; Table 4).

**Table 4:** Linear paramedian measurements with an angulation of 0°, 10°, 20°, 30°with the mean values (Mean), standard deviation (SD), minimal (Min) and maximum (Max) values, 95% CI, skewness and kurtosis in [mm]

	Mean [mm] (95% CI)	SD [mm]	Min [mm]	Max [mm]	Skewness	Excess Kurtosis
Point 1l 0°	12.18 (4.7; 19.66)	3.82	2.21	22.40	-0.255	-0.128
Point 1l 10°	11.57 (2.95; 20.18)	4.39	1.97	23.68	-0.233	-0.492
Point 1l 20°	10.55 (1.3; 19.79)	4.72	1.83	20.29	-0.196	-1.081
Point 1l 30°	9.18 (0.26; 18.09)	4.55	1.73	19.71	0.050	-1.149
Point 1r 0°	12.25 (4.9; 19.6)	3.75	1.06	22.62	0.038	0.067
Point 1r 10°	11.81 (3.45; 20.17)	4.27	0.75	21.90	-0.350	-0.345
Point 1r 20°	10.38 (1.47; 19.3)	4.55	0.76	20.10	-0.281	-0.979
Point 1r 30°	9.18 (0.1; 18.26)	4.63	0.74	20.80	0.018	-1.151
Point 2l 0°	9.26 (2.71; 15.82)	3.35	2.51	21.07	0.513	0.329
Point 2l 10°	9.63 (2.58; 16.67)	3.59	2.47	21.86	0.374	-0.096
Point 2l 20°	10.3 (2.78; 17.83)	3.84	2.57	20.93	0.075	-0.591
Point 2l 30°	11.0 (3,59; 18.42)	3.78	2.19	18.95	-0.279	-0.633
Point 2r 0°	9.23 (2.4; 16.07)	3.49	2.17	24.25	0.694	1.176
Point 2r 10°	9.65 (2.24; 17.06)	3.78	1.89	22.44	0.489	0.122
Point 2r 20°	10.23 (2.58; 17.87)	3.90	1.69	20.99	0.175	-0.565
Point 2r 30°	10.79 (3.26; 18.32)	3.84	1.66	19.05	-0.195	-0.783
Point 3l 0°	6.34 (1.26; 11.43)	2.59	1.12	15.94	0.080	1.068
Point 3l 10°	6.58 (0.95; 12.22)	2.87	1.13	16.51	0.888	1.019
Point 3l 20°	7.21 (0.57; 13.67)	3.34	1.18	18.02	0.965	0.709
Point 3l 30°	7.88 (0.73; 15.03)	3.65	1.28	18.33	0.689	-0.128
Point 3r 0°	6.38 (1.29; 11.46)	2.59	1.51	14.32	0.696	0.417
Point 3r 10°	6.59 (1.03; 12.16)	2.84	1.55	15.55	0.822	0.515
Point 3r 20°	7.21 (0.42; 14,0)	3.46	1.63	19.06	0.972	0.701
Point 3r 30°	7.93 (0.47; 15.39)	3.81	1.75	19.06	0.713	-0.273

A significant difference between age groups was found in model point 1r/1l. Children aged 13–16 years had a significantly higher MD than those aged 4–12 years (regression coefficient = 1.60; *p* = 0.02). No significant difference was found for 17–20-year-olds (*p* = 0.07) (Table 5). In the model point 2r/2l/point 3r/3l a significant difference between the age groups was found (*p* < 0.001). In all models a significant difference was found for sex, teeth and angulation (*p* < 0.001), but not for side (*p* > 0.05) (Table 5).

**Table 5:** Mixed regression models: variation in angulation point 1r/1l and point 2r/2l/point3r/3l depending on various influencing factors (age group, sex, teeth, side, angle, point) as additional independent variables with regression coefficient, 95% confidence interval and p-value. The effect of increasing angles on the measurement at point 1 is diametrically opposite to the effect of increasing angles at points 2 and 3. To handle this, we analyzed the two subgroups (point 1 and points 2/3) using two separate models, as these are easier to interpret than one large model including interaction terms

Point 1r/1l [mm]			Coefficient	95% CI		*p*-Value	
Parameter	Groups	Number [n]		Lower Limit	Upper Limit		
intercept			13.05	11.72	14.38	< 0.001	
age group	Reference: 4–12	33					
[years]	13–16	42	1.60	0.28	2.91	0.02	
	17–20	109	1.08	-0.08	2.24	0.07	
sex	Reference: male	122					
	female	62	-1.81	-2.71	-0.90	< 0.001	
teeth	Reference: no teeth	79					
	teeth	105	-2.15	-3.01	-1.30	< 0.001	
side	Reference: left	184					
	right	184	-0.04	-0.36	0.29	0.83	
angle	Reference: 0°	184					
	10°	184	-0.53	-0.99	-0.07	0.03	
	20°	184	-1.75	-2.21	-1.29	< 0.001	
	30°	184	-3.04	-3.50	-2.58	< 0.001	
Point 2r/2l/Point 3r/3l [mm]			Coefficient	95% CI		p-Value	
Parameter				Lower Limit	Upper Limit		
intercept			6.67	5.52	7.82	< 0.001	
age group	Reference: 4–12	33					
[years]	13–16	42	1.95	0.83	3.07	0.001	
	17–20	109	2.26	1.2	3.32	< 0.001	
sex	Reference: male	122					
	female	62	-1.94	-2.76	-1.11	< 0.001	
teeth	Reference: no teeth	79					
	teeth	105	2.62	1.92	3.32	< 0.001	
side	Reference: left	184					
	right	184	0.02	-0.1	0.13	0.79	
angle	Reference: 0°	184					
	10°	184	0.31	0.15	0.47	< 0.001	
	20°	184	0.91	0.75	1.07	< 0.001	
	30°	184	1.60	1.44	1.75	< 0.001	
point	Reference: 2r and 2l						
	3r and 3l	184	-3.01	-3.12	-2.90	< 0.001	

## Discussion

The aim of this study was to digitally analyze the safe “T-zone” of the hard palate, as defined in previous studies, in terms of bone volume and thickness to determine the insertion site and angle for orthodontic mini-implants. Palatal bone thickness was assessed both volumetrically and linearly, taking into account different potential implant insertion angles. Bone thickness was greatest at the level of the first maxillary premolars and decreased from anterior to posterior at the level of the first maxillary premolars and decreased from anterior to posterior (Table 2; Fig. 4). These results support the findings of previous studies [30–33]. An average length of 7–9 mm and a diameter of 2–2.3 mm are recommended for successful placement of orthodontic mini-implants to achieve adequate primary stability and secure intraosseous anchoring of the mini-implants [16, 17]. The results showed that these recommendations for bone thickness were not available in the posterior maxilla (ROI 3r/ROI 3l), where the mean measurements were 3.52 ± 1.13 mm and 3.44 ± 1.16 mm for the right and left sides, respectively (Table 2). Therefore, based on these results, insertion in this area cannot be recommended as it cannot be considered safe. The results demonstrated that the region with the greatest bone thickness was the anterior palate around the first premolars (ROI 1r/ROI 1l), with mean measurements of 10.44 ± 2.53 mm and 10.40 ± 2.52 mm, respectively (Table 2). These results are consistent with previous studies, where the thickest bone zone was also located in the anterior region [18, 30, 33, 34]. This study also measured bone thickness at different angles, simulating an anteriorly inclined insertion angle during orthodontic mini-implant placement. In the anterior region (point 1r/1l), angles of 20° and 30° run the risk of injuring the roots of the incisors, as the results showed that in 57% of the patients the roots of the anterior teeth were located within this measurement area, mimicking an orthodontic mini-implant. A high angulation (20° or 30°) resulted in a notable reduction in available bone thickness at point 1r/1l, which can be attributed to the proximity to the root of the incisor (regression coefficient = -3.04 (30°)) (Table 5). Therefore, an implant insertion angle of 0° or 10° corresponds to the recommendations for “safe” mini-implant insertion to avoid root damage while maintaining adequate bone thickness of 12.25 ± 3.75 mm (0°) and 11.81 ± 4.27 mm (10°) for point 1r (Table 4; Fig. 4). At point 2r/2l, corresponding to the level of the first premolars, a bone thickness of 9.23 ± 3.49 mm (0°) and 9.65 ± 3.78 mm (10°) was observed, respectively. However, a more pronounced angulation resulted in a notable increase in bone thickness, 10.23 ± 3.90 mm (20°) and 10.79 ± 3.84 mm (30°) (Table 4; Fig. 4). In contrast, the measured points in the region between the first and second premolars (point 3r/3l) achieved a lower bone thickness with a mean linear measurement of 6.34 ± 2.59 mm (Table 4; Fig. 4). Bone thickness in the posterior region of the palate increases significantly (*p* < 0.001) with greater angles of angulation. Even though the regression coefficients for 10° and 20° are significant, they can be considered less relevant for the clinician (regression coefficient 0.31 and 0.91 respectively) (Table 5). In the most posterior area, a 30° angulation is recommended to achieve maximum bone thickness (MD 3r = 7.93 ± 3.81 mm) (Table 4; Fig. 4). The statistical analysis also demonstrated a significant increase in bone thickness for 30° in this region, which is relevant for clinical considerations (regression coefficient = 1.60) (Table 5). From this it can be concluded that angulation is relevant at certain positions with close anatomical orientation to the anterior teeth. Regarding angulation, the results are in agreement with those of Becker et al. [18]. For bone thickness, the statistical analysis of the variables showed a significant difference for sex (*p* < 0.001), but not for age (*p* > 0.05). (Table 3). Female patients had on average 1.12 mm less bone thickness than male patients in all ROI sections (Table 3). With regard to angulation, a significantly lower bone thickness (*p* < 0.001) was also observed in female patients compared to male patients of the same age (regression coefficient= -1.81 and − 1.94) (Table 5). There were no significant differences between age subgroups (Table 3), confirming the findings of previous studies [18, 30, 32]. There was a significant difference in angle measurement at point 1r/1l between those aged 4–12 and those aged 13–16 (*p* = 0.02), but no significant difference between those aged 13–16 and those aged 17–20 (*p* = 0.07) (Table 5). This may be due to the fact that the maxilla stops growing at around 15 years of age [35]. Especially in younger patients, the palate is the preferred insertion site due to a significantly higher success rate compared to other insertion sites [36, 37]. To represent all age groups, the patients included in this study were younger (16.03 ± 3.78 years) than in comparable studies in the literature to represent all age groups [33]. In contrast, Chhatwani et al. found significant differences across all age groups in a slightly older cohort of patients [29]. Figure 4 shows that bone thickness tends to increase with age, although no statistically significant relationship can be demonstrated. This is particularly evident in the measurements at 0° and 10°. At the 30° angulation, there is also an increase in bone thickness with age, with point 2r/2l showing higher values than point 1r/1l. This is due to the proximity of the incisor roots as described above (Fig. 4; Tables 4 and 5). The statistical analysis showed a notable divergence in bone thickness when the teeth extended into the measured area. The conducted investigation yielded a significant reduction (*p* < 0.001) in bone thickness 2.15 mm (regression coefficient = -2.15) (Table 5). This reduction in bone thickness should be considered by the treating clinician for subsequent therapy.

For preoperative diagnosis prior to mini-implant placement, panoramic or periapical radiographs are appropriate for interradicular placement, and a lateral cephalogram is recommended for paramedian palatal placement [38–41]. However, two-dimensional images have limitations such as distortion and magnification, which can lead to inaccurate measurements and misinterpretation of existing space due to overlapping roots [38, 42–45]. Three-dimensional imaging modalities, such as CBCT, can image bone thickness, displaced teeth and anatomical structures [38, 46, 47]. Although modern CBCT scanners are now available with low-dose protocols that significantly reduce the radiation dose while still providing clinically acceptable images, their use should be considered according to the clinical indication and the “as low as diagnostically acceptable” (ALADA) principle, especially in younger patients [48–50]. The aim of this study was therefore to use a large cohort of patients to provide clinicians with information on which implant positions and angulations are “safe” when 3D images are not available. For this reason, the present study used pre-existing CBCTs obtained with a justified indication. To assist clinicians, the ROI was defined along the teeth [18]. Only patients with complete dentition and no asymmetries were selected to reduce inaccuracies.

Several methods of measuring bone quantity have been described in the literature. In this study, the palate was measured using both the “classic” linear point method, as in many previous studies, and a modern three-dimensional method that represents the volume and surface area of the palate [18, 31–33]. Seidel et al. established this method by combining CBCT scans with intraoral scans to assess palatal soft tissue thickness in the maxilla [51]. In addition to Seidel et al., there are other studies in the literature that have already established our method [51–55]. As the “classical” linear measurement only considers single points and these are usually placed at a distance of 2–3 mm, the data obtained is limited as it can be distorted by irregular palatal structures [29, 32]. In the area-based analysis of maxillary bone thickness performed here, bone thickness was measured at each individual point of the palatal STL mesh, then averaged over the areas and displayed in a color-coded distance map. (Fig. 3b). This means that several hundred points were acquired. This provides more information about the anatomical bone thickness of the palate than point measurements in the CBCT slices. The color-coded map shows that bone thickness decreases from anterior to posterior as well as in the medial area of the palatal suture, as shown in previous studies [18, 34]. Anatomical structures such as the nerve canal or the roots of the incisors are also visible (Fig. 3d). A 3D volumetric analysis of the palate (in mm^3^) for the orthodontic mini-implant placement has not been performed in the literature before, so a direct comparison of the findings with previous results was not possible.

The virtual definition of points and ROIs allows a high level of comparability between individuals in this study. Due to the large number of patients in this study (*n* = 184), the results are conclusive and comparable to other studies. Nevertheless, a high individual variability was observed as in previous studies [18, 56]. The deviation was particularly high in the anterior palatal region, where the roots of the incisors protruded into the examined area in 57% of cases and did not protrude in 43% of cases. In Chang et al., the roots of the maxillary teeth protruded into the anterior measurement area in 33% of cases (7 of 21 patients) [30]. This also explains why the SD decreases steadily from anterior to posterior (ROI 1r/1l SD = 2.53/2.52; ROI 2r/2l SD = 2.05/2.09; ROI 3r/3l SD = 1.13/1.16) (Table 2).

Angulation has been shown to play a critical role in the primary stability and therefore success rate of orthodontic mini-implants [57]. To date, there is little evidence that bone thickness has been measured at different angulations to simulate angled implant placement. Becker et al. also investigated palatal bone thickness at different insertion angles [18]. They used single linear measurements from cross-sectional views, similar to other studies [30, 34, 58]. However, Becker et al. also included angulation to simulate mini-implant placement, and because the measurement sites were also examined 3 mm and 6 mm paramedian along the teeth as in this study, the results are directly comparable. Different angles were chosen, anterior as in the present study and posterior (0°, 10°, 20°, 30° and posterior − 0°, -10°, -20°, -30°). They defined a “safe zone” that extended from the first premolars to the contact point between the first and second premolars. In this area, the bone quantity measurements in this study were also greater than 6.5 mm, except at point 3l (6.34 ± 2.59 mm) (Table 4). Accordingly, the paramedian zone defined by Becker et al. at the level of the first premolar can be considered safe in agreement with the results of the present study. In contrast to the “T-zone” defined by Becker et al., which also defines the median area as safe, the present study showed frequent proximity to the nerve canal [18]. Naya-Imai et al. showed that in 56% (84/150) of patients the nerve canal partially or completely extended into the area between the first and second premolar [59]. In order to protect this important structure, the area 3 mm paramedian at the level of the first premolar can be defined as a “safe zone”. It is recommended that medial insertion be avoided in children and adolescents as it may have a negative effect on the transverse development of the maxilla [59–61].

One statistical limitation of the study is that the assumption of normal distribution of residuals of our mixed models was violated. To validate the results of the mixed models, we performed equivalent non-parametric tests (Mann-Whitney U test and Wilcoxon signed-rank test, respectively, where appropriate). To avoid pseudoreplications that would artificially increase sample size and type I error, we averaged all statistically dependent measurements of bony palates, except where tested between dependent groups. Detailed results are provided in the appendix. Most significances found in the mixed models could be reproduced, with the exception of the difference of measurements with angle 0° and 10° in the model for point 1r/1l and the significant difference of the measurements between age groups 4–12 and 13–16 years at points 2r/2l, 3r/3l. This can have several reasons. The power of the non-parametric tests simply could be too small to detect these differences, but of course it may also be the case that the results of the mixed models are not valid here because of violated assumptions. We nevertheless chose to show the results of the mixed models because all trends (if not significant) were visible in the non-parametric analysis and this approach allowed us to include several interesting factors in few models and therefore allowed for a concise and clear presentation and discussion of results.

Furthermore, no explicit assessment of reliability or precision was conducted for this evaluation, as the method of analysis utilized is an established approach that has been demonstrated to be highly accurate [51, 52]. Prior research has evaluated the technique employed in this investigation, confirming that the CBCT image spatial resolution and the resulting 3D models fall within a clinically acceptable range of less than 300 μm [62].

It cannot be precluded that bone with lower density was integrated in the measurements of this study, but according to Marquezan et al., bone density does not play a significant role in the primary stability of mini implants, but mainly the compacta thickness of 1 mm or greater [1, 63]. Other studies have shown that cortical thickness is an important determinant of implant success. In particular, it has been found that the load on the mini-implants is even less with bicortical anchoring than with monocortical anchoring. This approach can effectively minimise the risk of implant fracture [64, 65]. Further clinical studies are needed to correlate the bone quality and stability/load, ideal mini-implant position and angle, and with treatment success.

Another limitation of this study was that the angle was only measured at single points in the area of the ‘T-zone’ already defined by Becker et al. and not over the entire ROI.

As in previous studies, a large individual variability was observed [18, 56]. It would be interesting to investigate the relationship between maxillary bone thickness and skeletal type. Anatomical structures such as the nerve canal can pose a risk of injury during implantation [59]. Further studies should therefore investigate the exact location of the nerve channel in three dimensions in order to provide the clinician with a recommendation for avoiding this risk and thus making the implantation safer.

The present study focused exclusively on younger patients. However, it would be beneficial to conduct a similar investigation with older individuals.

The use of guided implant placement and digital planning is becoming increasingly popular for conventional dental implants, as it has been reported in the literature to be even more accurate than freehand placement [66, 67]. In the case of orthodontic mini-implants, Wilmes et al. investigated digital planning for orthodontic applications in two clinical cases [68]. However, a larger patient cohort would be required to evaluate this technique for orthodontic mini-implant placement in the future. The volumetric and linear 3D evaluation method presented in this study can provide a high quality methodology for further research.

## Conclusion and clinical relevance

The results of this clinical study showed that the palatal paramedian region at the level of the first premolar is the ideal site for placement of orthodontic mini-implants, as determined by precise three-dimensional measurements: this region had the highest bone volume and linear thickness measurements. To maximize the usable bone thickness, the angulation of the implants should be chosen according to the insertion region. In the anterior region of the palate, a reduced angulation of 0° or 10° is recommended in order to avoid injury to the roots of the incisors. Conversely, in the posterior region of the palate, an increase in angulation is recommended to achieve a longer anchoring distance. The research also showed differences in palatal bone volume between males and females. These results may help to improve the safety of orthodontic mini-implant placement in practice.

## Electronic supplementary material

Below is the link to the electronic supplementary material.

Supplementary Material 1

## Data Availability

All data generated or analyzed during this study are included in this published article.

## Declarations

### Reporting guideline

STROBE checklist.

## Abbreviations

2D: Two-dimensional
3D: Three-dimensional
95% CI: 95% confidence interval
AA: Analysis area
ALADA: As low as diagnostically acceptable
CAD: Computer-aided design
CBCT: Cone beam computed tomography
DICOM: Digital Imaging and Communications in Medicine
HU: Hounsfield unit
l: Left
MD: Mean distance
r: Right
ROI: Regions of interest (ROI)
STL: Standard Tessellation Language
TADs: Temporary anchorage devices
Vol: Volume
