# Supplementary material for: Three-dimensional digital imaging analysis of the palatal bone thickness for orthodontic mini-implant insertion – determination of the safe zone and angulation
Source: BMC Oral Health. 2024 Nov 28;24:1448. doi: 10.1186/s12903-024-05229-y (PMC11603675; doi:10.1186/s12903-024-05229-y)
Supplement: Supplementary file 1 — Supplementary Material 1 [file 12903_2024_5229_MOESM1_ESM.docx]

Supplementary file

Validation of significant findings of the mixed linear models was done using non-parametric statistical tests (Mann-Whitney U test and Wilcoxon-signed rank test, respectively). In the following, the mixed regression models described in Table 3 and Table 5 will be referred to as models 1, 2 (Table 3) 3, and 4 (Table 5).

Additional file 1 (File format: jpg. – Joint Photographic Experts Groupe)

Model1


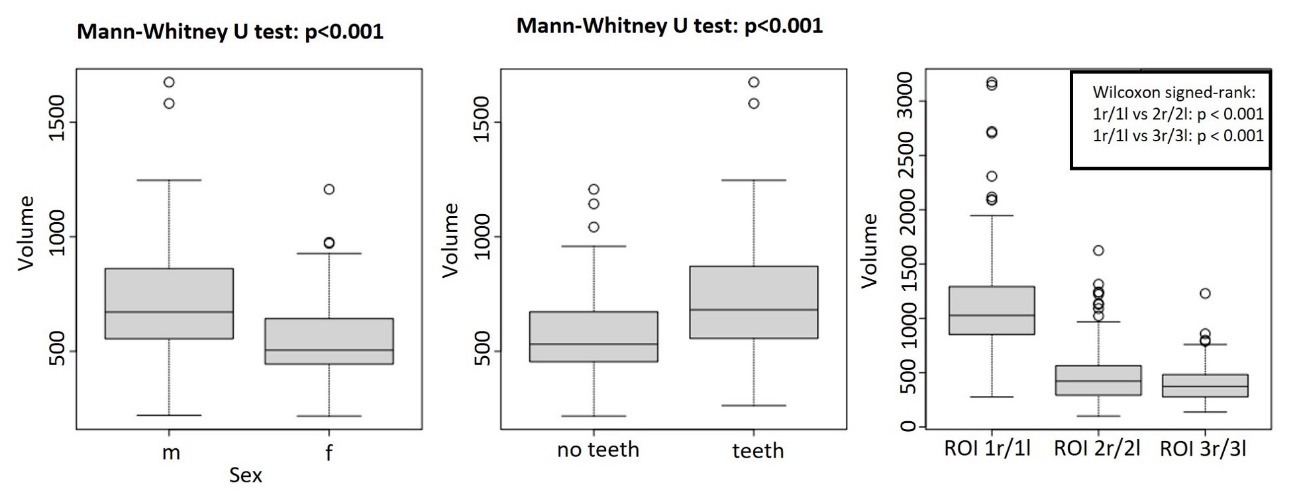


Additional file 1: Analysis of volume
To avoid pseudoreplications, volume and distance measurements were averaged to obtain one measurement per patient (analysis of sex and teeth, Mann-Whitney U test) or one measurement per ROI of each patient (comparison of ROI measurements, left and right were averaged; Wilcoxon-signed rank test).

Additional file 2 (File format: jpg. – Joint Photographic Experts Groupe)

Model 2


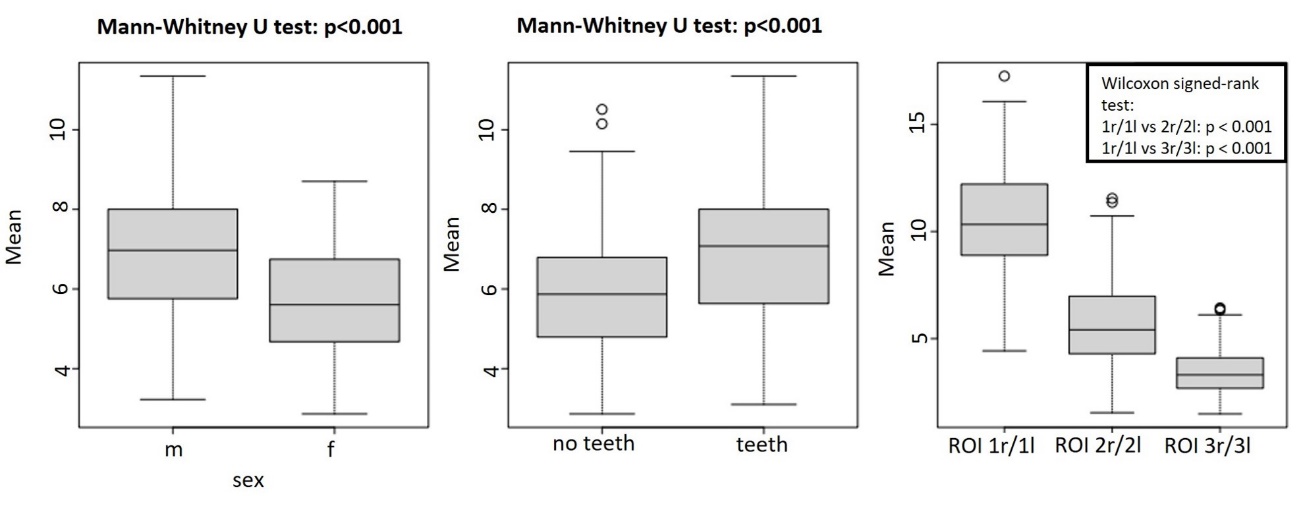


Additional file 2: Analysis of mean distance
To avoid pseudoreplications, volume and distance measurements were averaged to obtain one measurement per patient (analysis of sex and teeth, Mann-Whitney U test) or one measurement per ROI of each patient (comparison of ROI measurements, left and right were averaged; Wilcoxon-signed rank test).

Additional file 3 (File format: jpg. – Joint Photographic Experts Groupe)

Model 3


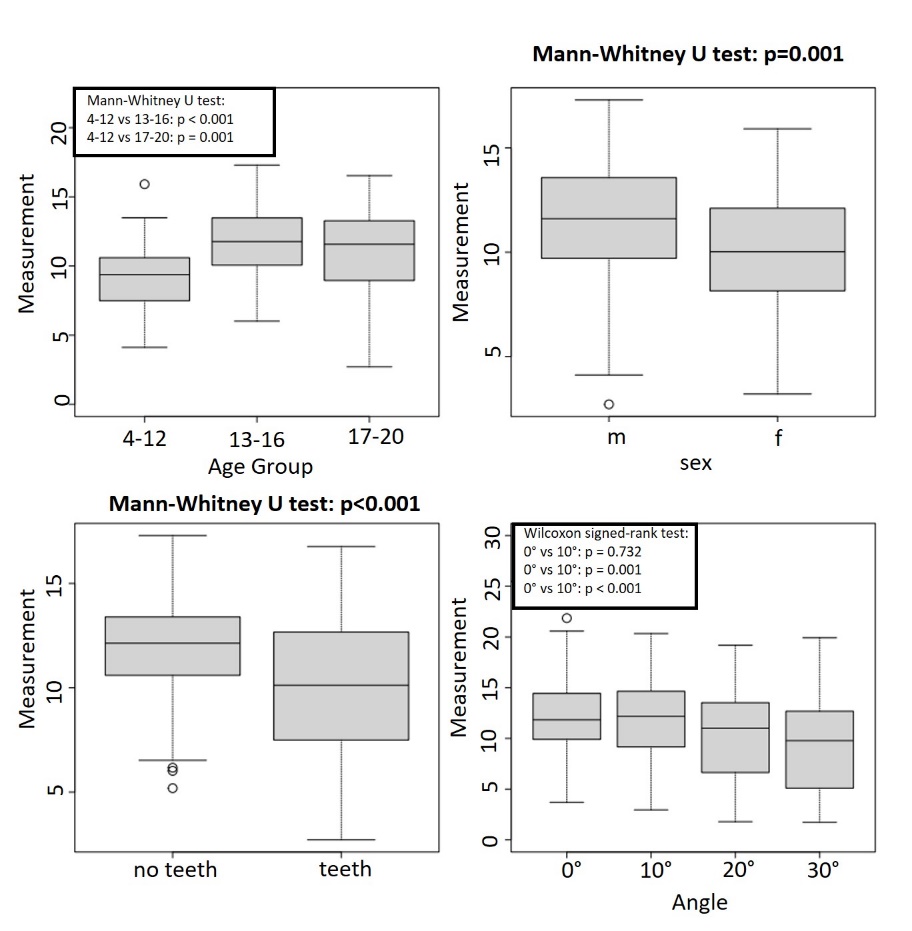


Additional file 3: Analysis of angulation I
To avoid pseudoreplications, measurements were averaged to obtain one measurement per patient (analysis of age group, sex, and teeth; Mann-Whitney U test) or one measurement per angle of each patient (comparison of angles, left and right were averaged; Wilcoxon-signed rank test). To validate model 3, the subgroup of measurements for point 1r/1l was used.

Additional file 4 (File format: jpg. – Joint Photographic Experts Groupe)

Model4


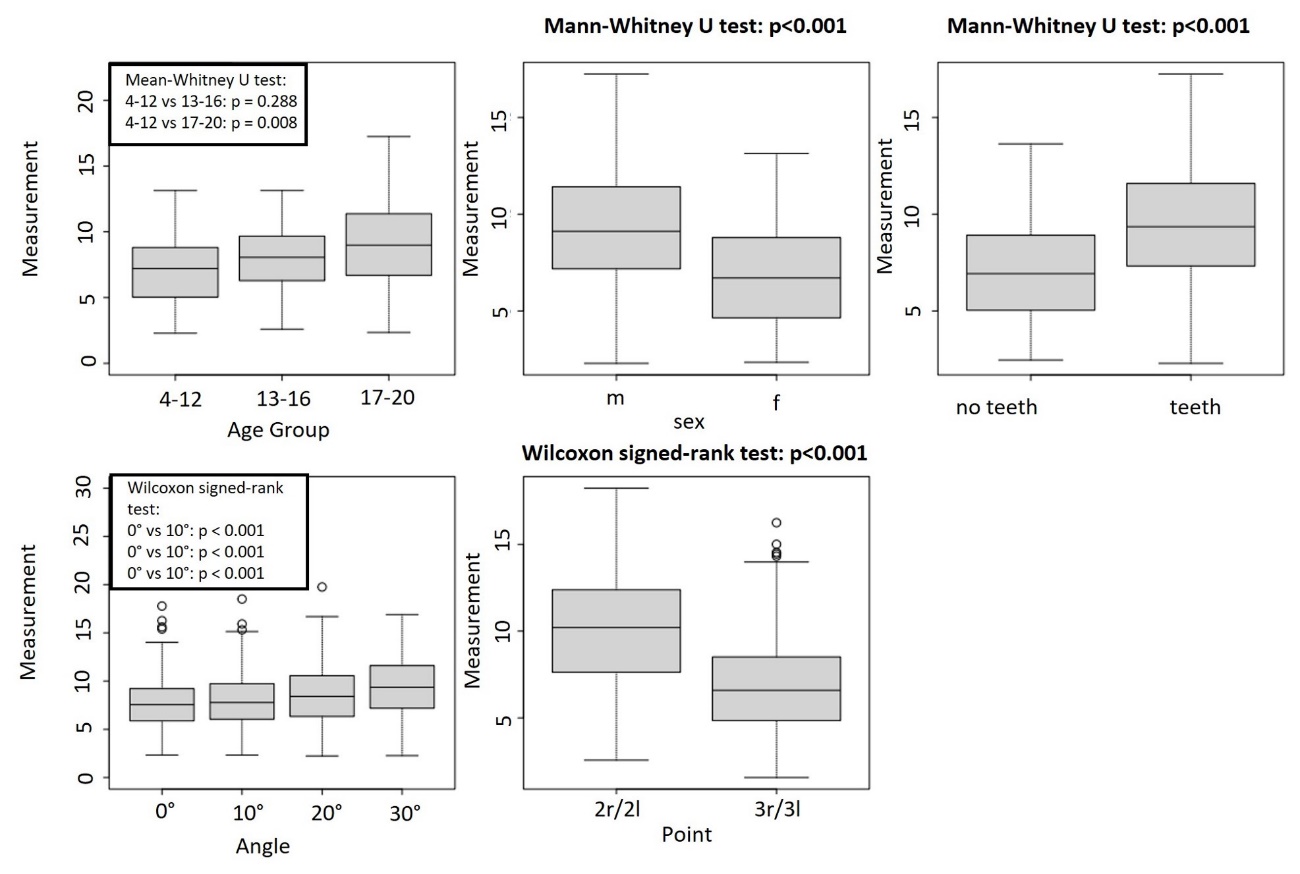


Additional file 4: Analysis of angulation II
To avoid pseudoreplications, measurements were averaged to obtain one measurement per patient (analysis of age group, sex, and teeth; Mann-Whitney U test) or one measurement per angle of each patient (comparison of angles, left and right were averaged; Wilcoxon-signed rank test). To validate model 3, the subgroup of measurements for point 1r/1l was used. To validate model4, the subgroup of measurements for points 2r/2l and 3r/3l was used. Additionally to the steps performed to validate model 3, measurements were averaged to obtain one measurement per point per patient and compared (Wilcoxon signed-rank test) to validate model 4.
